# Effects of SPARCL1 on the proliferation and differentiation of sheep preadipocytes

**DOI:** 10.1080/21623945.2021.2010901

**Published:** 2021-12-07

**Authors:** Cheng Xiao, Hai Guo Jin, Li Chun Zhang, Jian Qiang Liu, Ming He, Hui Hai Ma, Yong Sheng Yu, Yang Cao

**Affiliations:** a Institute of Animal Biotechnology, Jilin Academy of Agricultural Sciences, Jilin, Gongzhuling, China; b Institute of Animal Husbandry and Veterinary Medicine, Jilin Academy of Agricultural Sciences, Jilin, Gongzhuling, China

**Keywords:** *SPARCL1*, preadipocyte, proliferation, differentiation, apoptosis, sheep

## Abstract

Important candidate genes that regulate lipid metabolism have the potential to increase the content of intramuscular fat (IMF) and improve meat quality. *Secreted protein acidic and rich in cysteine like 1*(*SPARCL1*) is a secreted glycoprotein with important physiological functions and is involved in the proliferation and differentiation of various cells. However, the role of the *SPARCL1* gene in sheep preadipocytes and its regulatory mechanism is still unclear. In this study, we explored the effect of *SPARCL1* on the proliferation and differentiation of sheep preadipocytes. The results showed that the expression level of the *SPARCL1* gene is higher in fat tissue than in other tissues, and the gene was significantly increased on the 6^th^ day of preadipocyte differentiation. In the preadipocyte proliferation stage, interference of *SPARCL1* gene reduced cell viability and increased cell apoptosis. In preadipocyte differentiation stage, *SPARCL1* overexpression significantly inhibited lipid droplets accumulation and triglyceride content by increasing *Wnt10b, Fzd8, IL6*, and *β-catenin* and inhibiting *PPARγ, C/EBPα, LPL*, and *IGF1* genes expression, whereas *SPARCL1* deficiency significantly promoted cell differentiation by inhibiting *β-catenin* and increasing *GSK3β, PPARγ, C/EBPα*, and *LPL*. The results of this study suggest that *SPARCL1* plays a negative role during preadipocyte differentiation and may become a novel target for regulating preadipocyte differentiation and improving IMF.

**Abbreviations:** **IMF**: Intramuscular fat ***SPARCL1***: Secreted protein acidic and rich in cysteine like 1 ***PPARγ***: Peroxisome proliferator-activated receptor γ ***C/EBPα***: CCAAT/enhancer-binding protein-α ***LPL***: Lipoprotein lipase ***IGF1***: Insulin-like growth factor 1 ***Wnt10b***: Wnt family member 10B ***Fzd8***: Frizzled class receptor 8 **IL6**: Interleukin 6 ***β-catenin***: Catenin beta interacting protein 1 ***GSK3β***: Glycogen synthase kinase 3 beta ***LRP5/6***: Low-density lipoprotein receptor-related protein 5/6

## Introduction

Mutton is becoming more and more popular with consumers in China. Small Tail Han sheep is a unique breed in China, with high fecundity and strong viability, and it was widely bred in Northeast China to meet the local demand for mutton in the past [1]. However, the growth rate and meat quality of Small Tail Han sheep are not as good as foreign sheep breeds, and cannot meet the current consumer market demand, therefore it is urgent to improve its meat quality. Many factors influence meat quality, among them increasing the content of intramuscular fat (IMF) can significantly improve the taste, tenderness, colour, and quality of meat by promoting the formation and differentiation of adipocytes and the accumulation of triglycerides in lipid droplets [2,3]. Therefore, it is essential to detect new target genes that promote adipocyte differentiation.

Adipocyte formation is a complex physiological process involving a large number of genes, non-coding RNAs, growth factors, and signal pathways [4,5]. The transformation of preadipocytes into mature adipocytes requires two stages of preadipocyte proliferation and differentiation [6]. The proliferative phase of preadipocytes, although short, is a necessary process for adipocyte formation, and a variety of adverse factors can lead to apoptosis and prevent preadipocyte maturation. The peroxisome proliferator-activated receptor gamma (PPARγ) a transcription factor, specifically expressed in adipose tissue, plays a decisive role in preadipocyte differentiation [7]. The transcription factor CCAAT/enhancer-binding protein-alpha (*C/EBPα*) also plays a vital role in adipocyte differentiation [8]. *PPARγ* positively regulates *C/EBPα* and co-initiates preadipocyte differentiation [9]. Lipoprotein lipase (*LPL*) is a key enzyme of fat deposition, hydrolysing triglycerides and promoting lipoprotein uptake [10]. In addition, insulin-like growth factor 1 (*IGF1*) also promotes adipocyte proliferation and differentiation [11]. These genes have emerged as biomarkers for detecting preadipocyte differentiation. Many signalling pathways are also involved in adipocyte differentiation. Several studies have found that the Wnt/β-catenin signalling pathway plays an important role in adipocyte formation and that the *β-catenin* gene has a key regulatory function as a second messenger [12]. *β-catenin* can affect downstream genes (*PPARγ* and *C/EBPα*) to regulate adipocyte differentiation [13].

Secreted protein acidic and rich in cysteine like 1 (SPARCL1), a member of the SPARC family, is a glycoprotein that mediates cell-matrix interactions and is involved in many physiological processes, including cell adhesion, proliferation, differentiation, migration, and maturation [14], as well as being an important regulator of cellular metabolism. In recent years, *SPARCL1* has been extensively studied in cancer and could be a potential target for cancer therapy [15], and it has recently been reported that *SPARCL1* regulates adipogenesis in mice [16], but the exact mechanism is unknown and there are limited studies in sheep lipid metabolic processes. Therefore, the present study explores the potential effects of the *SPARCL1* gene on the proliferation and differentiation of sheep preadipocytes and its mechanism, providing a new target for increasing IMF content and a new research direction for other fields.

## Materials and methods

### Experimental animal

The experimental animals were three two-month-old and three six-month-old healthy male Small Tail Han sheep from the Institute of Animal Biotechnology, Jilin Academy of Agricultural Sciences. The groin adipose tissue of two-month-old sheep was used to extract preadipocytes. Heart, muscle, small intestine, stomach, liver, duodenum, and fat tissues of six-month-age male sheep were isolated for qPCR validation. Animal experiments were performed following animal use protocols approved by the Committee for the Ethics of Animal Experiments (AWEC2017A01, 9 March 2017).

### Preadipocyte isolation, culture, and differentiation

The adipose tissue was washed with PBS containing 1% penicillin/streptomycin solution (Sigma-Aldrich, St. Louis, MO, USA), and the connective tissue and blood clots in the adipose tissue were removed using sterile tweezers. The pure tissue was cut into mm^3^ pieces with surgical scissors, and the blocks were digested collagenase II(Sigma-Aldrich) in a water bath at 37°C for 1 h, mixing every 15 minutes, and the undigested tissue and miscellaneous cells were filtered out using 200 mesh(75 μm) and 400 mesh filters(38 μm), and then the supernatant was removed by centrifuging at 1500 rpm for 15 min to obtain preadipocyte. Next, the preadipocytes were cultured using a complete medium containing DMEM-F12 (Sigma-Aldrich), 10% foetal bovine serum (Gemini Bio-Products, Woodland, CA, USA), and 1% penicillin/streptomycin solution in 60-mm Petri dishes (Corning, Corning, NY, USA) in a 37 ^o^C and 5% CO_2_ incubator, and change the culture medium every 48 h. When the cells overgrew the petri dish, some preadipocytes were digested with trypsin (Sigma-Aldrich) to subculture, and the remaining preadipocytes were induced by exogenous inducer I (10 mg/mL insulin (Sigma-Aldrich), 1 mM dexamethasone (Sigma-Aldrich), 0.5 mM isobutylmethylxanthine (Sigma-Aldrich) and complete medium) and inducer II solution (10 mg/mL insulin (Sigma-Aldrich) and complete medium) into mature adipocytes. The inducer I and II have respectively cultured the cells for 48 h and then exchanged a fresh complete medium to culture the cells until becoming mature adipocytes.

### Oil red O staining

When preadipocytes are differentiated into mature adipocytes, the intracellular lipid droplets can be stained red by oil red O dye to verify the maturation of the adipocytes. Adipocytes were washed three times with PBS and fixed with 4% paraformaldehyde (Sangon Biotech Co., Ltd., Shanghai, China) in a closed environment for 30 min, then washed three times with PBS and stained with 0.5% oil red O (Sangon Biotech) in an incubator for 30 min. Finally, the cells were washed a time with PBS and observed and photographed under the microscope. Isopropyl alcohol can dissolve intracellular lipid droplets, and the absorbance value at 490 nm detects lipid droplet content.

### Detection of triglyceride content

Intracellular triglyceride content was determined by the triglyceride detection kit (Prilax, Beijing, China), following the supplier´s instructions. Differentiated cells were washed three times with PBS and digested with trypsin (Sigma-Aldrich) and centrifuged at 1500 rpm for 15 min. Lysis solution was then added to the adipocytes pellet at room temperature for 10 minutes, followed by heating at 70°C for 10 minutes and centrifuging at 2000 rpm for 5 minutes. The supernatants were detected triglycerides by measuring ODs at 550 nm wavelength by an enzyme-labelled instrument and calculating triglyceride content.

### SPARCL1 *interference sequence design*

Based on the sheep *SPARCL1* mRNA sequence available on the NCBI website (accession number: XM_004009982.3), we designed the following small interference sequences and a negative control sequence: si-1217-*SPARCL1*(sense:5ʹ-CCAAGGAGCCUUCUAACAATT-3ʹ; antisense: 5ʹ-UUGUUAGAAGGCUCCUUGGTT-3ʹ), si-1937-*SPARCL1* (sense:5ʹ-GCACUGACAAUCAGACCUATT-3ʹ; antisense: 5ʹ-UAGGUCUGAUUGUCAGUGCTT-3ʹ), si-2244-*SPARCL1* (sense:5ʹ-CCUUCUCUUAAGAGACUUUTT-3ʹ; antisense: 5ʹ-AAAGUCUCUUAAGAGAAGGTT-3ʹ), Negative control (sense:5ʹ-UUCUCCGAACGUGUCACGUTT-3ʹ; antisense: 5ʹ-ACGUGACACGUUCGGAGAATT-3ʹ). These sequences were synthesized by Shanghai GenePharma Gene Co. Ltd. (Shanghai, China). We verified the optimal siRNA sequence by cell transfection and qPCR for further experiments.

### *Construction of* SPARCL1 *overexpression plasmid*

Based on the sheep *SPARCL1* mRNA sequence available on the NCBI website, we designed primer sequences to clone the CDS region of the *SPARCL1* gene. The primers were synthesized by Suzhou Jin Weizhi Co. Ltd. (Suzhou, China). PEX4 vector and the restriction endonucleases XhoIII and EcoRI were used to construct recombinant plasmid completed by Shanghai GenePharma Gene Co. Ltd. pEX4 vector was used as the negative control.

### Cells transfection

When the cells grew to 70% of the culture dish, we started transfection of plasmids or interference sequences to the cells with Lipofectamine 2000 (Thermo Fisher Scientific, Waltham, MA, USA) transfection reagent. Lip2000 needs to be placed at room temperature for 5 min in advance, and 100 pmol siRNA and 5 uL lip2000 were added to 200 uL Opti-MEM (Invitrogen, Carlsbad, CA, USA) forming a mixed solution and placed at room temperature for 10 min. Each 6-well plate was spiked with 200 ul of the above mixture and 1.8 ml of fresh culture medium for 6 h. After this, we replaced the mixture with the complete culture medium, then detected the transfection efficiency by qPCR at 48^th^ h. FuGENE (Roche Applied Science, Indianapolis, IN, USA) was a transfection reagent of the overexpression vector. 5 uL FuGENE solution was added to 200 uL Opti-MEM in an Eppendorf tube and placed at room temperature for 5 min, 3 mg plasmid was added to 200 uL Opti-MEM in another Eppendorf tube and placed at room temperature for 5 min. The two solutions were mixed well and placed at room temperature for 20 min. Each 6-well plate was spiked with 200 ul of the above mixture and 1.6 ml of fresh culture medium for 6 h. After this, we replaced the mixture with a complete culture medium. The recombinant plasmid contains green fluorescent protein, so we used fluorescence microscopy to detect the efficiency of cell transfection and qPCR to detect the results of plasmid transfection at 48^th^ h.

### Cell counting kit

The plasmid or siRNAs were transfected to the cells in 96-well plates for 48 h, then removed the culture medium and added the 10 mL/well of Cell Counting Kit-8(CCK8, Beyotime, China) solution to each well in a 37°C incubator for 4 h. The OD value at 450 nm was read for each well and then the cell survival rate was calculated. Untransfected cells were used as a negative control and CCK8 alone was used as a low OD value.

### Apoptosis detection by flow cytometry

After 48 hours of cell transfection, cells were digested with 0.25% trypsin (Gibco BRL, Life Technologies) without EDTA for 5 min, then collected in Eppendorf tubes and centrifuged at 300 x g and 4°C for 5 min, next were washed twice with PBS and centrifuged at 300 x g and 4°C for 5 min, after which we resuspended the cells with 100 uL of apoptosis detection kit buffer (US Everbright®Inc, Suzhou, China) with 4 uL of annexin V and 5 uL of PI working solution and incubated it in darkness at room temperature for 15 min. Finally, 400 uL PBS was added to each tube and immediately evaluated under flow cytometry. Annexin V emits spectra at 530 nm (FITC channel) and 617 nm (PI channel). Untransfected cells were used as a negative control.

### RNA extraction and qPCR assay

We used TRIzol reagent (Thermo Fisher Scientific) to extract cellular total RNA extracts on days 2, 4, 6, 8, and 10 during cellular proliferation and differentiation, and the experimental steps are described in the instructions. The cells growing to 75% of the culture dish were on the 2nd day; The cells growing to 100% of the culture dish and added with induction solution I were on the 4th day; The cells were replaced with induction solution II on the 6th day; Replaced with fresh culture medium were on the 8th day; many cells differentiated into the mature adipocytes on the 10th day. Monitoring RNA degradation and contamination of cells and tissues using 1% agarose gels, followed by measurement of RNA concentration using a Quawell Q5000 spectrophotometer (Quawell Technology, San Jose, CA, USA). Next, we reversed the RNA to the cDNA using a reverse transcription kit (Takara, Japan). The reverse transcription system consisted of 2 μL of 5× PrimeScript RT Master Mix, 500 ng of total RNA, and RNase Free ddH_2_O up to 10 μL, and reaction conditions were 37 ^o^C for 15 min, 85 ^o^C for 5 sec, and storage at 4 ^o^C, and then the reaction products were diluted 10 times by double-distilled water. The quantitative fluorescent PCR detects the relative expression of mRNA levels in a Roche LightCycler® 480 (Roche Applied Science), and the Glyceraldehyde-3-Phosphate dehydrogenase (*GAPDH*) gene was used as an internal control. The reaction system consisted of 10 μL of 2× Light Cycler 480 SYBR Green I Master, 8 μL of ddH_2_O, 1 μL of cDNA, and 0.5 μL of sense and antisense primers. The reaction conditions were 40 cycles of 95 ^o^C for 5 min, 95 ^o^C for 10 s, 60 ^o^C for 15 s, and 72°C for 20 s, followed by 95 ^o^C for 5 s, 65 ^o^C for 1 min, and 40 ^o^C for 10 s. The number of experimental repetitions was three times. The primer sequences are in Table 1.

**Table 1.** Primer sequences for a quantitative real-time polymerase chain reaction

Gene	Oligo	Primer sequence	Product size	GenBank No.
*Sparcl1*	Forward Primer Reverse Primer	5ʹ ATCCAGCATCTTGTCCTCCTAC 3ʹ 5ʹ CCTTAGAGGAAACTGGGTCACT 3’	205 bp	XM_004009982.3
*PPARγ*	Forward Primer Reverse Primer	5ʹ CCGTGGACCTTTCTATGATGG 3ʹ 5ʹ TACAGGCTCCACTTTGATTGC 3’	193 bp	NM_001100921.1
*C/EBPα*	Forward Primer Reverse Primer	5ʹ AAGCCAAGAAGTCCGTGGAC 3ʹ 5ʹ AGCACCTTCTGTTGCGTCTCC 3’	126 bp	NM_001308574.1
*GAPDH*	Forward Primer Reverse Primer	5ʹ TCCACGGCACAGTCAAGG 3ʹ 5ʹ CACGCCCATCACAAACAT 3’	228 bp	NM_001190390.1
*LPL*	Forward Primer Reverse Primer	5ʹ AGGACACTTGCCACCTCATTC 3ʹ 5ʹ AGCCAGTCCACCACGATGA 3’	189 bp	XM_027963889.1
*IGF1*	Forward Primer Reverse Primer	5ʹ CAGTCACATCCTCCTCGCA 3ʹ 5ʹ TACATCTCCAGCCTCCTCAG 3’	249 bp	NM_001009774.3
*Wnt10b*	Forward Primer Reverse Primer	5ʹ GCAGTCCACGAGTGTCAGCA 3ʹ 5ʹ AGCCAGCATGGAGAAGGAAA 3ʹ	141 bp	XM_004023070.4
*FZD 8*	Forward Primer Reverse Primer	5ʹ CTCCATCTGGTGGGTGATCCT 3ʹ 5ʹ CGCAAGTTGTCCAGGCTCTG 3’	216 bp	XM_027976634.1
*β-catenin*	Forward Primer Reverse Primer	5ʹ CTGGCAGCAGCAGTCTTACCT 3ʹ 5ʹ ACTCATACAGGACTTGGGTGGT 3ʹ	125 bp	XM_027972680.1
*GSK-3β*	Forward Primer Reverse Primer	5ʹ ATAATCAAGGTCCTGGGAACAC 3ʹ 5ʹ TCCAGCAGACGGCTACAAA 3ʹ	161 bp	NM_001129740.1
*IL6*	Forward Primer Reverse Primer	5ʹ GTCCACTGGGCACATAACTTATG 3ʹ 5ʹ CGTTCAAGCCGCATAGCCA 3ʹ	216 bp	NM_001009392.1

### Western blot analysis

We used a protein extraction kit (Solarbio, Shanghai, China) to extract total cellular protein extracts on days 2, 4, 6, 8, and 10 during cellular proliferation and differentiation. The cells were washed with PBS and treated with lysis solution for 30 min on ice and centrifuged at 12,000 rpm at 4°C for 30 min. The protein concentration was determined BCA solution, and lysate regulates the protein concentration to make the same concentration of total protein in each group. The supernatant with proteins was then boiled at 100°C for 10 minutes to denature proteins and mixed with SDS-PAGE buffer (Beyotime, Jiangsu, China). Preparing separation and concentrated gels using the PAGE Gel Fast Preparation kit (EpiZyme, Shanghai, China). After the gel solidification, 20 ug of proteins and 5 uL of protein markers (Thermo Fisher Scientific) were injected into the gel hole at 80 voltages for 20 min. Voltage was changed to 120 V for 40 min after proteins reached the separation gel. We transferred the proteins from the gel to a 0.45 mm Immobilon poly-vinylidene difluoride membrane (Millipore, Bedford, MA, USA) at 200 mA for 1 h, after which the PVDF membrane was washed with TBST buffer for 5 min and sealed with 5% skimmed milk powder blocking solution for 2 h. The membrane was then washed three times with TBST buffer for 5 minutes each. The membrane was incubated with antibodies and shaken overnight at 4°C. On the second day, the membrane was washed three times with TBST buffer for 5 minutes each, incubated with secondary antibodies (anti-rabbit or anti-mouse IgG-HRP, 1:5000) at room temperature for 2 h, and washed three times with TBST buffer for 5 minutes each. The chromogenic reaction of proteins used ECL hypersensitive photoluminescence solution (Pripril, Beijing, China). The protein bands data were detected using the ChemiScope 6000 Touch imaging system (Clinx Science Instruments, Shanghai, China), and were quantified using the ImageJ software. β-actin was used as an internal reference protein. The antibody dilution ratio was in Table 2.

**Table 2.** The information of antibodies used in Western blot

Gene	Description	Dilution	Source, Number
*Sparcl1*	Rabbit polyclonal antibody	1:1000	Boster, DZ1007
*PPARγ*	Rabbit polyclonal antibody	1:1000	Bioss, bs-0530 R
*C/EBPα*	Rabbit polyclonal antibody	1:1000	Bioss, bs-1630 R
β*-catin*	Rabbit polyclonal antibody	1:5000	Bioss, bs-1571 R
*LPL*	Rabbit polyclonal antibody	1:1500	Bioss, bs-1973 R
*IGF1*	Rabbit polyclonal antibody	1:1000	Bioss, bs-0227 R
*Wnt10b*	Rabbit polyclonal antibody	1:1000	Bioss, bs-3662 R
*FZD 8*	Rabbit polyclonal antibody	1:1000	Bioss, bs-13,219 R
β-catenin	Rabbit polyclonal antibody	1:1000	Bioss, bs-1165 R
*GSK-3β*	Rabbit polyclonal antibody	1:1000	Bioss, bs-0028 R
*IL6*	Rabbit polyclonal antibody	1:1000	Bioss, bs-4587 R

### Statistical analysis

All the results are expressed as means ± SEM. Comparisons between two groups using an unpaired, two-tailed Student’s t-test. Multiple group comparisons used one-way ANOVA. Data analysis was in Graph Pad Prism 6.0 software. The original image and data are in the supplementary file. The statistical significance levels were set at *P* < 0.05.

## Result

### SPARCL1 *gene expression is the highest in adipose tissue, with significant expression at day 6 of cell differentiation*

**Figure 1.**
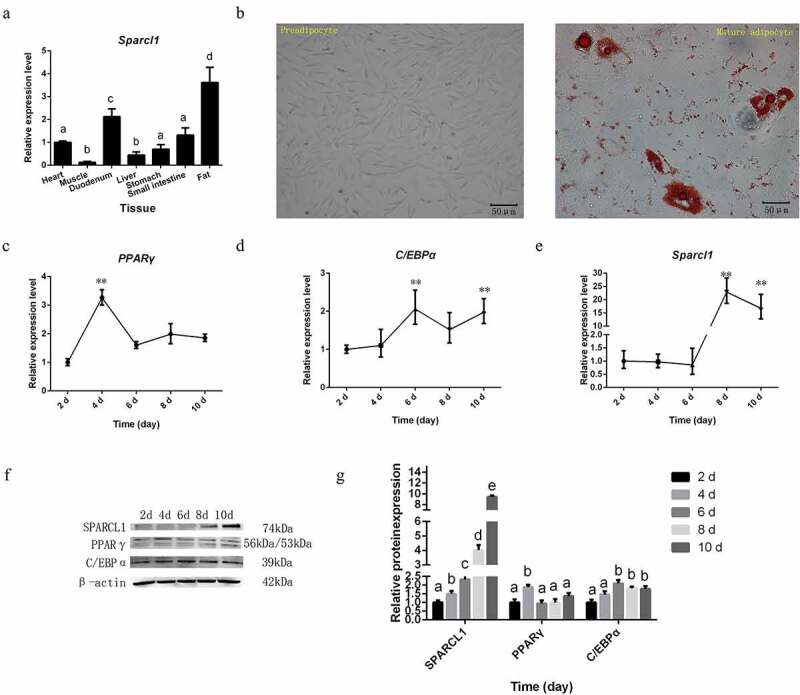
*SPARCL1* and adipogenic marker genes expression pattern during sheep preadipocyte differentiation. (a) *SPARCL1* gene expression in the different tissues. The first set of data in the figure is named ‘a’. Lowercase letters different from a represent significant data differences between groups (*P* < 0.05), named ‘b, c, and d’ in that order, and the same lowercase letters represent non-significant data differences(*P* > 0.05). (b) Sheep preadipocytes and oil red O staining. The red dots are lipid droplets inside the cell stained red by oil red O (200×; scale bar:50 μm). (c-e) *PPARγ, C/EBPα* and *SPARCL1* expression patterns in preadipocyte differentiation (**P < 0.01). (f) The protein expression levels of SPARCL1, PPARγ and C/EBPα during preadipocyte differentiation (2,4,6,8,10 days). (g) Densitometric analyses of the Western blots. The first set of data in the figure is named ‘a’. Lowercase letters different from a represent significant data differences between groups (*P* < 0.05), named ‘b, c, and d’ in that order, and the same lowercase letters represent non-significant data differences(*P* > 0.05)

*SPARCL1* gene expression was highest in adipose tissue (*P* < 0.01; Figure 1(a)). As shown in Figure 1(b), we successfully isolated, cultured, and differentiated sheep preadipocytes. Lipid droplets in mature adipocytes were stained red by Oil Red O. We examined marker genes during preadipocyte differentiation as well as target gene expression patterns. During preadipocyte differentiation, the expression level of *PPARγ* was the highest on the 4th day (*P* < 0.01; Figure 1(c)); *C/EBPα* gene high expression occurred on the 6th day, after which expression decreased and increased (*P* < 0.01; Figure 1(d)); *SPARCL1* gene has significantly increased on the 6th and 10th days (*P* < 0.01; Figure 1(e)), and the qPCR results of these genes were consistent with Western blot results Figure 1(f,g).

### Detection of transfection efficiency

**Figure 2.**
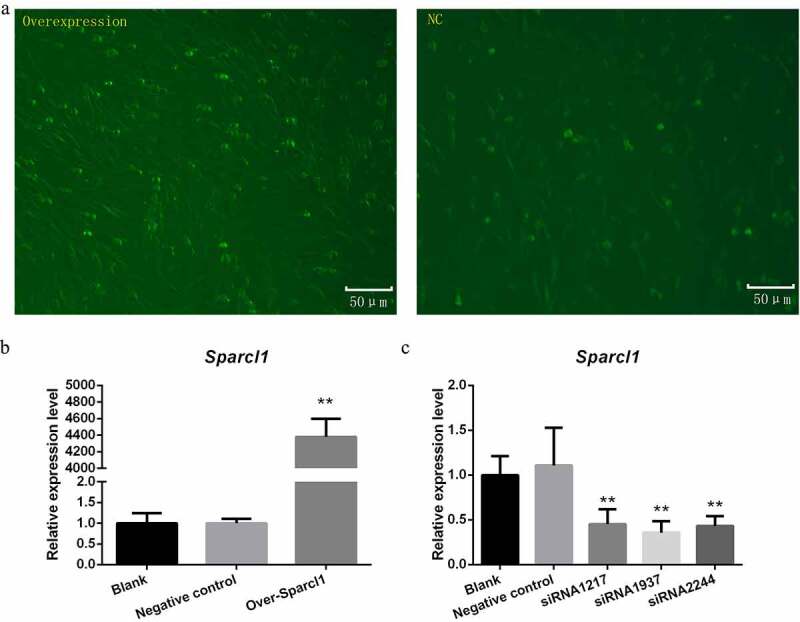
Validation of transfection efficiency of overexpression plasmid and interference sequence. (a) Determination of the transfection efficiency of overexpression plasmid (200×; scale bar:50 μm). Green dots are green fluorescent proteins expressed by the plasmids transfected into the cells. Green dots in the overexpression were more than NC group (**P < 0.01). (b) Expression levels of *SPARCL1* gene 48 hours after transfection with the overexpression plasmid (**P < 0.01) (c) Expression levels of *SPARCL1* gene 48 hours after transfection with the siRNA (**P < 0.01)

We synthesized overexpression plasmids and interference sequences to detect the effect of the *SPARCL1* gene on preadipocyte proliferation and differentiation. Post-transfection fluorescence assay of the cells also indicated high transfection efficiency Figure 2(a). Forty-eight hours after plasmid transfection of the cells, we verified the efficiency by qPCR. As shown in Figure 2(b), the experimental group’s overexpression plasmid increased the *SPARCL1* gene by more than 4000-fold (*P* < 0.01). As shown in Figure 2(c), among the three interfering sequences, siRNA1937 had the best inhibiting effect and was used for further experiments (*P* < 0.01).

### SPARCL1 *inhibition increases* apoptosis and reduces viability on sheep preadipocytes proliferation phase

**Figure 3.**
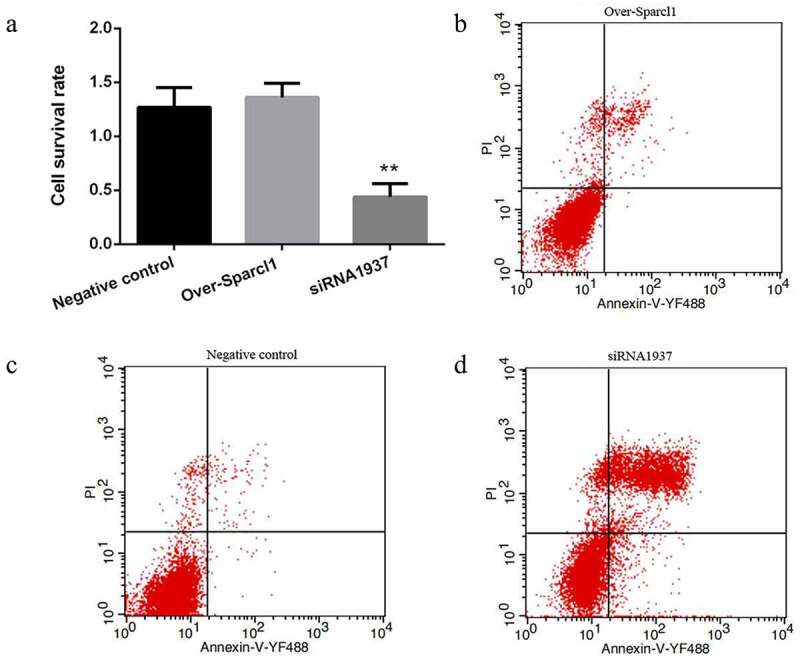
*SPARCL1* Inhibition decreases cell survival rate and increases apoptosis. (a) Cell counting kit-8 (CCK8) shows that siRNA1937 significantly decreases cell survival rate(**P < 0.01). (b-d) Determination of apoptosis by flow cytometry. *SPARCL1* inhibition significantly increases apoptosis(*P* < 0.01); *SPARCL1* overexpression do not lead to apoptosis(*P* > 0.05). Red dots represent cells. Flow cytometry chart is divided into four areas, the lower left is living cells, the lower right is early rising apoptotic cells, the top right is apoptotic cells, the top left is dead cells. The number of detected cells is 10,000

We performed CCK8 assays on the 4^th^ day to determine the effect of *SPARCL1* on the sheep preadipocytes proliferation. The results showed that interference with SPARCL1 inhibited cell proliferation and reduced cell viability (*P* < 0.01), but overexpression had no significant effect on cell proliferation and viability (*P* > 0.05; Figure 3(a)). We found by flow cytometry assay that *SPARCL1* interference increased apoptosis and thus decreased cell proliferation and viability (*P* < 0.01), while *SPARCL1* overexpression had no significant effect on apoptosis (*P* > 0.05; Figure 3(b-d)).

### SPARCL1 *is a negative regulator of preadipocytes differentiation in sheep*

**Figure 4.**
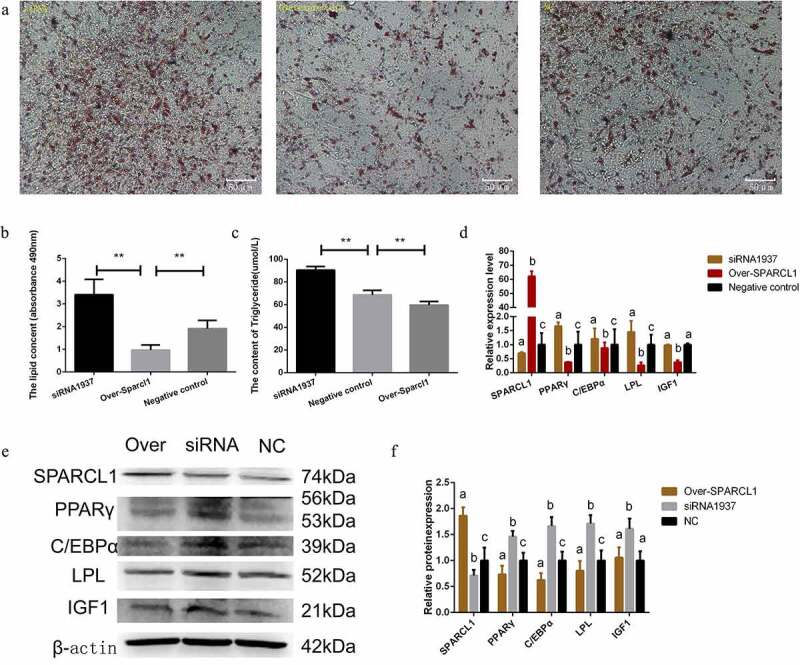
*SPARCL1* gene is a negative regulator of adipocyte differentiation (a and b) effect of oil red O staining to detect *SPARCL1* on preadipocyte differentiation(200×; scale bar:50 μm); *SPARCL1* Inhibition significantly increases promotes adipocyte lipid droplets concent (**P < 0.01); *SPARCL1* overexpression inhibits adipocyte lipid droplets concent (**P < 0.01); (b) The figure digitized the oil red O diagram and the absorbance value at 490 nm. (c) The content of triglyceride in the negative control, overexpression and inhibition groups was determined on the 12th day of differentiation. *SPARCL1* inhibition significantly increases promotes the content of adipocyte triglyceride(**P < 0.01); *SPARCL1* overexpression inhibits the content of adipocyte triglyceride (**P < 0.01); (d) Inhibition and overexpression with the *SPARCL1* gene affect the mRNA expression of *PPARγ, C/EBPα, LPL, IGF1*. (e) Inhibition and overexpression with the *SPARCL1* gene affect the protein expression of PPARγ, C/EBPα, LPL, IGF1. (f) Densitometric analyses of the Western blots. The first set of data in the figure is named ‘a’. Lowercase letters different from a represent significant data differences between groups (*P* < 0.05), named ‘b, c, and d’ in that order, and the same lowercase letters represent non-significant data differences(*P* > 0.05)

We examined intracellular triglyceride content and expression level of marker genes to assess the effect of *SPARCL1* on preadipocyte differentiation. The results showed that inhibition of *SPARCL1* significantly increased triglyceride content and intracellular lipid droplets on the 12^th^ day (*P* < 0.01; Figure 4(a-c)) and significantly increased the expression of the adipogenic marker genes *PPARγ, LPL*, and *C/EBPα* (*P* < 0.05; Figure 4(d-f)). In contrast, overexpression of *SPARCL1* significantly reduced triglyceride content and intracellular lipid droplets on the 12^th^ day(*P* < 0.01; Figure 4(a-c)) and significantly reduced *PPARγ, LPL, IGF1*, and *C/EBPα* expression (*P* < 0.05; Figure 4(d-f)). Thus, *SPARCL1* negatively regulates cell differentiation and triglyceride accumulation by regulating the expression of adipogenic marker genes.

### SPARCL1 *may regulate the Wnt/*β-catenin *pathway to affect preadipocyte differentiation*

**Figure 5.**
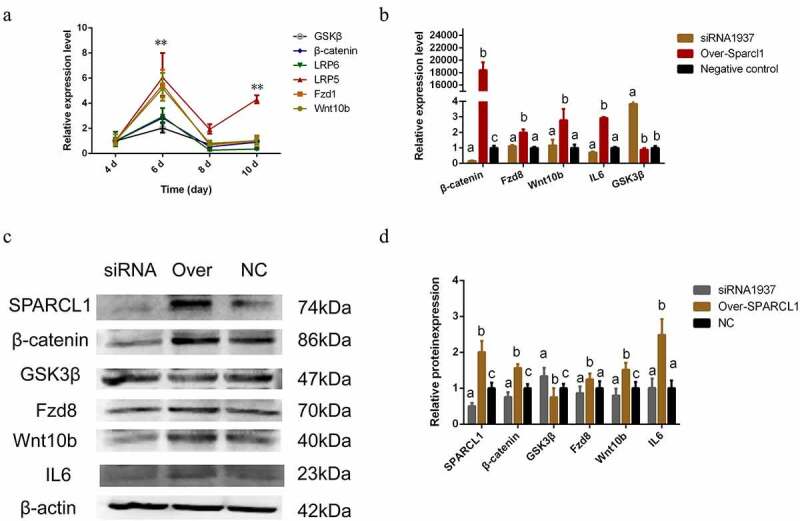
*SPARCL1* affects the Wnt/β-catenin pathway related genes (a) *GSK3β, FZD8, Wnt10b, β-catenin, IL6* expression patterns in preadipocyte differentiation; These genes expression is the highest at day 6(**P < 0.01). (b) Inhibition and overexpression with the *SPARCL1* gene affect the mRNA expression of *GSK3β, FZD8, Wnt10b, β-catenin, IL6*. (c) Inhibition and overexpression with the *SPARCL1* gene affect the protein expression of GSK3β, FZD8, Wnt10b, β-catenin, IL6. (d) Densitometric analyses of the Western blots. The first set of data in the figure is named ‘a’. Lowercase letters different from a represent significant data differences between groups (*P* < 0.05), named ‘b, c, and d’ in that order, and the same lowercase letters represent non-significant data differences(*P* > 0.05)

Studies showed that the *SPARCL1* gene may be associated with the Wnt/β-catenin pathway and has a role in cancer as well as other diseases, so we tested whether the *SPARCL1* gene can regulate the Wnt/β-catenin pathway affecting preadipocyte differentiation [17,18]. We examined the pattern of changes in key genes (*Wnt10b, β-catenin, LRP5/6, Fzd8*, and *GSK3β*) in the Wnt/β-catenin pathway during adipocyte differentiation to explore its possible mechanisms. As shown in Figure 5(a), The expression trends of these genes were similar, reaching a maximum on the 6^th^ day, after which the expression level decreased (*P* < 0.05). Based on an understanding of their expression patterns, we chose day 6 of cell differentiation to detect the effect of *SPARCL1* on these genes. The results showed that *SPARCL1* overexpression significantly increased *Wnt10b, Fzd8, β-catenin*, and *IL6*, whereas *SPARCL1* interference significantly decreased *β-catenin* and increased *GSK3β* (*P* < 0.05; Figure 5(b-d)). Therefore, *SPARCL1* may affect Wnt/β catenin pathway genes regulating *PPARγ, C/EBPα*, and *LPL* to affect preadipocyte differentiation.

## Discussion

Studies have shown that *SPARCL1* inhibits tumour cell growth and serves as a marker for cancer detection and a therapeutic target. As the direction of research has expanded, some reports have shown that the SPARCL1 gene has additional physiological functions, e.g, *SPARCL1* inhibits adipogenesis in 3T3-L1 cells [16]; SPARCL1 is highly upregulated in adipose tissue in patients with non-alcoholic steatohepatitis [B. 19, 20]. Also, we downloaded three GEO data (GSE97241, GSE90580, and GSE51905) associated with 3T3-L1 cell differentiation from the public data platform. It turned out that these data share a common differentially expressed gene which is *SPARCL1*, which also strongly suggests that *SPARCL1* may affect cellular adipogenesis. However, the function and regulatory mechanisms of lipid metabolism in *SPARCL1* are not known, particularly for sheep lipid metabolism, and we are therefore keen to conduct in-depth studies. In the present study, *SPARCL1* expression was the highest in the subcutaneous adipose tissue, suggesting that *SPARCL1* may be involved in the process of sheep adipogenesis. *SPARCL1* expression was unchanged by the addition of exogenous inducer and early preadipocyte differentiation. However, *SPARCL1* expression increased more than 20-fold on the 8th day. This implies that *SPARCL1* plays a role in late cell differentiation and in lipid metabolism. *PPARγ, C/EBPα* is known to be a key transcription factor for cell differentiation. *PPARγ* has a decisive role in the early stages of cell differentiation. When *PPARγ* is activated, it induces *C/EBPα* and targets to promote cell differentiation [21]. Thus, *PPARγ* expression was significant when exogenous inducers were added. Subsequently, *C/EBPα* expression was initiated. In the present study, the expression trends of *PPARγ* and *C/EBPα* were consistent with other reports. The results indicate that the experimental data are reliable and accurate.

Preadipocytes become mature adipocytes through proliferation and differentiation [22], so we explored the effect of *SPARCL1* genes on sheep preadipocytes at two different stages of proliferation and differentiation. First, we observed that *SPARCL1* expression was low in cell proliferation and did not alter cell viability and apoptosis rates. when *SPARCL1* expression was increased. However, some studies have shown that *SPARCL1* inhibits cancer cell proliferation [23]; *SPARCL1* overexpression inhibits renal cancer cell migration and invasion, which is different from our study, where overexpression of the *SPARCL1* gene did not affect sheep adipocyte proliferation [24]. It is possible that the adipocytes did not produce changes due to the regulation of other genes or factors, which needs to be explored more deeply, and this is where our present study is insufficient. *SPARCL1* interference reduced cell viability and increased the rate of apoptosis, yet in other studies, *SPARCL1* inhibited cancer cellular proliferation [25]. It is possible that adipocytes have different outcomes due to the presence of proliferation and differentiation and different mechanisms than cancer cells, a part we still need to investigate further. Our results suggest that interference with *SPARCL1* may reduce cell proliferation, whereas SPARCL1 overexpression does not affect cell proliferation.

At the stage of adipocyte differentiation, *SPARCL1* plays a negative role in preadipocyte differentiation and the results are consistent with other studies [16]. Because *PPARγ* and *C/EBPα* have a decisive effect on adipocyte differentiation, *SPARCL1* inhibition or overexpression can affect the expression of *PPARγ, C/EBPα, LPL*, and *IGF1*, thereby regulating intracellular triglyceride and lipid droplet content. What is the mechanism by which the *SPARCL1* gene regulates preadipocyte differentiation? Some studies have shown that *SPARCL1* can promote C2C12 cell differentiation [Y. 26], which is not contradictory to the present study, because both adipocytes and myogenic cells are derived from mesenchymal stem cells [27], and they are interconvertible, and some genes that can inhibit mesenchymal stem cells from becoming myogenic cells and promote adipocyte differentiation. Among them, WNT/*β-catenin* signalling pathway-related genes have similar functions, so we linked *SPARCL1* and WNT/*β-catenin* signalling pathways, and also some studies have shown that *SPARCL1* is associated with WNT/*β-catenin* signalling pathways [28]. Wnt signalling converts mesenchymal stem cells into preadipocytes but inhibits adipocyte differentiation [29]. Wnt signalling also improves muscle cell differentiation in different periods [D. 30]. Competition for wnt signalling exists between muscle cells and adipocytes [31]. *SPARCL1* has a similar function with Wnt/β-catenin signalling. Also, both SPARCL1 and Wnt are highly expressed in the cytoplasm [32], so we examined the effect of SPARCL1 on Wnt/β-catenin signalling. *Wnt10b* is lowly expressed in adipocyte differentiation, and overexpression of *SPARCL1* significantly increases *Wnt10b*, which is then enriched in membranes and binds *Fzd8* in the present study [33]. Wnt10b-containing polyprotein decreases the *GSK3β* and increases *β-catenin* suppresses *PPARγ* and *C/EBPα* [D. 34]. *GSK3β* translocates to the nucleus, phosphorylates *PPARγ* and *C/EBPα*, and promotes adipocyte differentiation [35]. In the present study, we were also able to observe that *SPARCL1* overexpression decreased *GSK3β, PPARγ, C/EBPα*. The results are consistent with Wnt/β-catenin signalling regulating adipocyte differentiation [36,37]. Although Wnt/β-catenin-related genes peaked at day 6 during preadipocyte differentiation, and *SPARCL1* gene expression started to rise at day 6, it did not affect the conclusion of this experiment, and the results just indicate that *SPARCL1* is one of the factors that can affect Wnt/β-catenin-related genes. Wnt/β-catenin is affected by a variety of factors during preadipocyte differentiation. *SPARCL1* can regulate Wnt/β-catenin-related genes to influence preadipocyte differentiation.

The present study is the first to show the role of *SPARCL1* in sheep adipocyte differentiation, and the study has practical implications. On the one hand, this study provides a new research direction to explore the regulatory mechanism of sheep preadipocyte differentiation. The newly identified regulatory genes can be used as molecular markers for screening sheep with good meat quality, and the application of genetic engineering technology can improve the meat quality of Small Tail Han sheep. On the other hand, *SPARCL1* may provide new therapeutic targets for metabolic diseases caused by obesity, which can help us better understand adipose differentiation and metabolism.

*SPARCL1* is involved in the process of sheep preadipocyte differentiation and plays a role in late differentiation. In addition, interference with *SPARCL1* leads to apoptosis. *SPARCL1* may be a negative regulator of adipocyte differentiation and lipid droplet accumulation. Thus, *SPARCL1* may be a new potential target for increasing IMF content in sheep.

## Supplementary Material

Supplemental Material

## Data Availability

The datasets used and analyzed during the current study are available from the corresponding author on reasonable request.
