# Supplementary material for: Effects of SPARCL1 on the proliferation and differentiation of sheep preadipocytes
Source: Adipocyte. 2021 Dec 7;10(1):658–69. doi: 10.1080/21623945.2021.2010901 (PMC8654481; doi:10.1080/21623945.2021.2010901)
Supplement: Supplemental Material [file KADI_A_2010901_SM9119.zip › supplementary/downloadFromZipFile2.pdf]

FIG1A-PCR-data-SPARCL1

Tissues	Relative expression level		
	Mean	+error	-error
Heart	1.000002	0.06086784	0.05737552
Muscle	0.1232794	0.04044167	0.03045194
Duodenum	2.123832	0.3422675	0.2947645
Liver	0.4454504	0.1348839	0.1035336
Stomach	0.7038484	0.196927	0.1538749
Small intestine	1.316466	0.3150193	0.254193
Fat	3.613349	0.671788	1.142970

FIG1C-PCR-data-PPAR γ

Days	Relative expression level		
	Mean	+error	-error
2 d	1.000002	0.1256692	0.1116396
4 d	3.264066	0.2775972	0.255839
6 d	1.602143	0.1226953	0.1139674
8 d	1.989186	0.362910	0.3339775
10 d	1.855831	0.1291171	0.1243333

FIG1D-PCR-data-C/EBP α

Days	Relative expression level		
	Mean	+error	-error
2 d	1.000023	0.111691	0.1004697
4 d	1.104480	0.4224444	0.3055694
6 d	2.061032	0.4959469	0.399754
8 d	1.515752	0.4507369	0.3474239
10 d	1.977074	0.3514987	0.2984399

FIG1E-PCR-data-SPARCL1

Days	Relative expression level		
	Mean	+error	-error
2 d	1.000000	0.3885393	0.2798187
4 d	0.9726549	0.2877597	0.2220625
6 d	0.856584	0.6278026	0.3622814
8 d	22.890340	5.308316	4.309041
10 d	16.718030	5.271169	4.007584

FIG1G-WB-data

Days	Protein bands gray value			
	SPARCL1	PPARγ	C/EBPα	β-actin
2d	10187	39834	24959	119114
4d	16066	79299	38430	126251
6d	25626	40424	56548	128827
8d	42251	40763	46572	122637
10d	99013	56127	45698	122733

FIG2B-PCR-data

Overexpression vector transfection	Relative expression level		
	Mean	+error	-error
Blank	1.000000	0.2454345	0.1970674
NC	0.9999977	0.1075885	0.09713759
OVER	4379.853000	217.372100	207.094000

FIG2C-PCR-data

siRNA transfection	Relative expression level		
	Mean	+error	-error
Blank	0.9999769	0.210966	0.1742123
NC	1.106983	0.4200267	0.3044922
SiRNA1217	0.451678	0.1680718	0.2298644
SiRNA1937	0.3568278	0.1291317	0.09481817
SiRNA2244	0.4302857	0.1127029	0.2683018

FIG3A-CCK8-data

Vector transfection	Cell survival rate		
	Mean	+error	-error
NC	1.270000	0.180000	0.180000
OVER	1.360000	0.130000	0.130000
SiRNA1937	0.440000	0.120000	0.200000

FIG3B- Apoptosis data-OVER
Gated Events: 10000
X Parameter: Annexin-V-YF488 (Log)
Quad Location: 18, 22
Quad Events % Gated
UL 302 3.02
UR 355 3.55
LL 9329 93.29
LR 14 0.14

FIG3C- Apoptosis data-NC
Gated Events: 10000
X Parameter: Annexin-V-YF488 (Log)
Quad Location: 18, 22
Quad Events % Gated
UL 181 1.81
UR 111 1.11
LL 9683 96.83
LR 25 0.25

FIG3D- Apoptosis data-siRNA
Gated Events: 10000

X Parameter: Annexin-V-YF488 (Log)

Quad Location: 18, 22

Quad Events % Gated

UL 559 5.59

UR 3374 33.74

LL 5618 56.18

LR 449 4.49

FIG4B- lipid concent-data

Vector transfection	The Lipid Concent		
	Mean	+error	-error
SiRNA1937	3.4082	0.6723	0.7214
OVER	0.9725	0.2351	0.3418
NC	1.1924	0.3658	0.2893

FIG4C- Triglyceride-data

Vector transfection	The Content Of Triglyceride		
	Mean	+error	-error
SiRNA1937	90.410000	3.170000	1.170000
NC	68.510000	4.060000	1.060000
OVER	59.570000	3.220000	1.220000

FIG4D-PCR-data

Vector transfection	NC			siRNA			OVER		
Genes	Mean	+error	-error	Mean	+error	-error	Mean	+error	-error
Sparcl1	1.000000	0.4104506	0.4476513	0.698985	0.03755294	0.03563827	62.106250	3.634839	3.433868
PPARγ	1.000000	0.4666648	0.4642852	1.655197	0.1362426	0.1343429	0.3728499	0.01879358	0.01789174
C/EBPα	1.000000	0.548910	0.5133563	1.207202	0.3720774	0.2844161	0.8806659	0.1968213	0.1608685
LPL	1.000000	0.3547671	0.2618658	1.445598	0.3964176	0.3111052	0.2654785	0.1025047	0.07395118
IGF1	1.000000	0.04306396	0.04128602	0.9816859	0.02788898	0.02711856	0.3771822	0.07007152	0.05909337

FIG4F-Protein-data

Groups	Protein bands gray value					
	PPARγ	C/EBPα	LPL	IGF1	β-actin	SPARCL1
over	156651	31886	43501	35309	141678	135261
siRNA	310803	84513	92259	53477	140881	62583
NC	211350	50535	53563	33004	140071	52568

FIG5A-PCR-data

Days	Wnt10b			Fzd1			LRP5		
	Mean	+error	-error	Mean	+error	-error	Mean	+error	-error
4 d	1.000000	0.5297513	0.346299	1.000000	0.1898109	0.1595303	1.000000	0.1501524	0.130550
6 d	5.193354	1.229706	0.9942767	5.451550	1.210811	0.9907597	6.062866	1.932509	1.465415
8 d	0.8122524	0.2276278	0.1778005	0.6877709	0.06908055	0.06277533	1.918528	0.4107467	0.3383152
10 d	1.028114	0.3060569	0.2358479	0.988514	0.4486352	0.3085847	4.306950	0.3303374	0.3068058

Days	LRP6			β-catenin			GSKβ		
	Mean	+error	-error	Mean	+error	-error	Mean	+error	-error
4 d	1.000000	0.7286699	0.4215205	1.000000	0.1122757	0.1009423	1.000000	0.2275209	0.185350
6 d	2.948539	0.6719846	0.5472614	2.834970	0.09000839	0.08723862	2.032610	0.4534312	0.3707295
8 d	0.2606165	0.03720656	0.0325584	0.5334161	0.0194389	0.01875541	0.7169776	0.09708522	0.08550683
10 d	0.364334	0.06573441	0.05568714	0.9012505	0.08168361	0.07489555	1.002313	0.3190615	0.2420203

FIG5B-PCR-data

Vector transfection	NC			siRNA			OVER		
Genes	Mean	+error	-error	Mean	+error	-error	Mean	+error	-error
β-catenin	1.000000	0.1445376	0.1262847	0.1584025	0.03150386	0.02627764	18411.680000	1262.137000	1181.167000
Fzd8	1.000000	0.07152545	0.06675105	1.113425	0.06819749	0.06483123	1.993133	0.191665	0.1684851
Wnt10b	1.000000	0.2156192	0.1773739	1.147565	0.3837844	0.3075214	2.786267	0.7118941	0.7983362
IL6	1.000000	0.06409641	0.06023553	0.7046604	0.05433359	0.05044404	2.927414	0.06270475	0.06072904
GSK3β	1.000000	0.1268446	0.1125662	3.828201	0.3290226	0.3029822	0.8796491	0.09645783	0.08692597

FIG5C-WB-data

Groups	Protein bands gray value						
	SPARCL1	β-CATENIN	GSK3β	FZD8	WNT10	IL6	β-actin
siRNA	18483	58175	91240	61669	68616	13112	151652
Over	74463	121132	51858	89731	131833	32440	152577
NC	37278	77829	69092	72092	87264	13110	153129
