# Supplementary figures and images for: Effects of SPARCL1 on the proliferation and differentiation of sheep preadipocytes
Source: Adipocyte. 2021 Dec 7;10(1):658–69. doi: 10.1080/21623945.2021.2010901 (PMC8654481; doi:10.1080/21623945.2021.2010901)

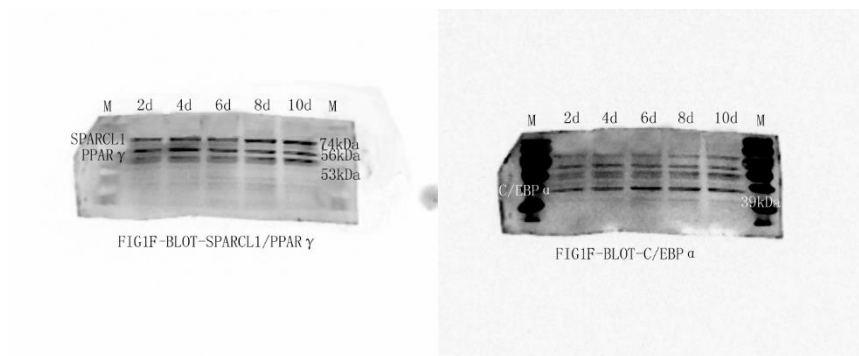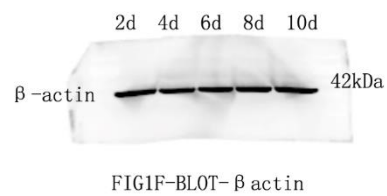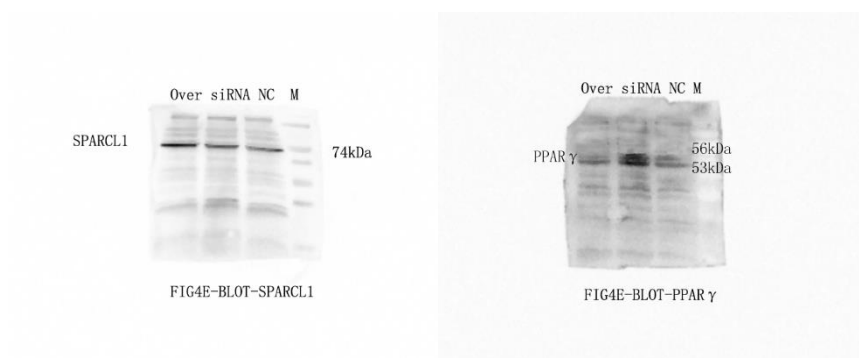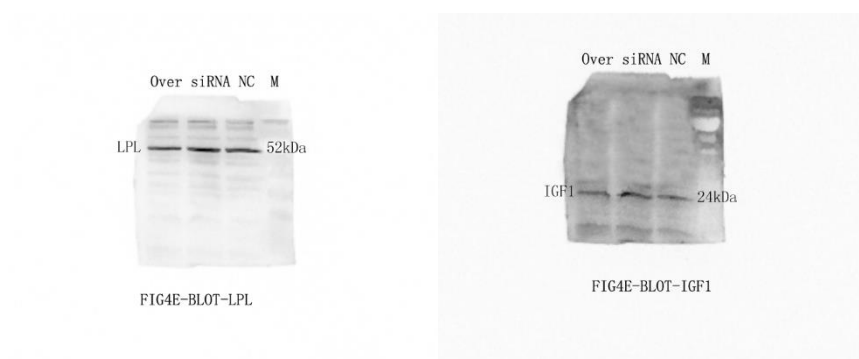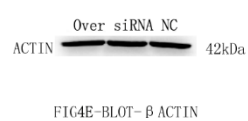

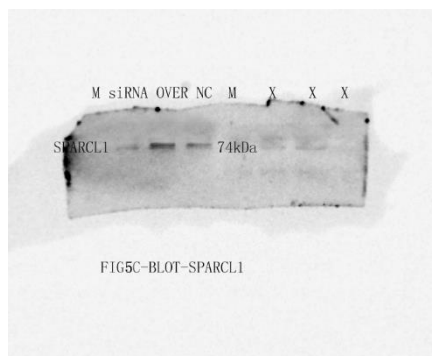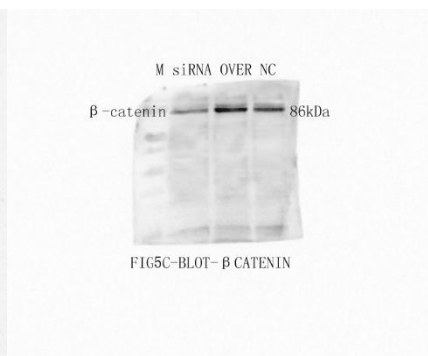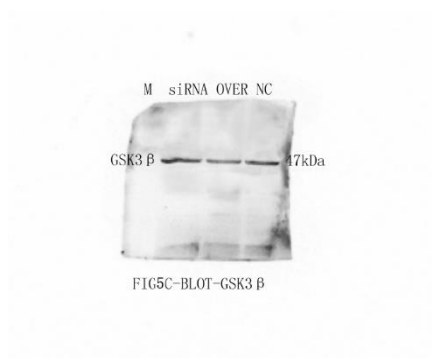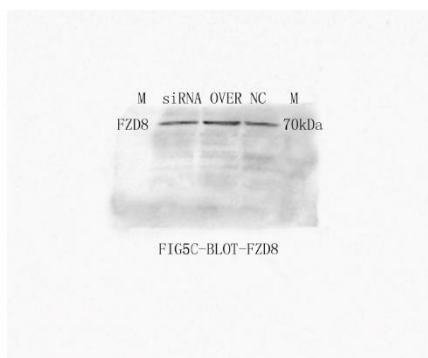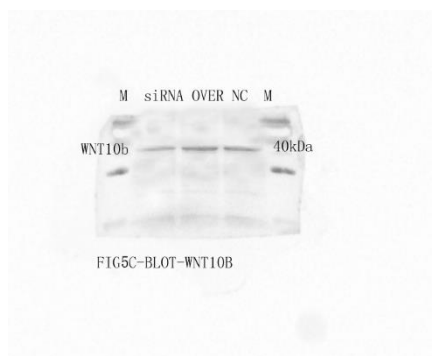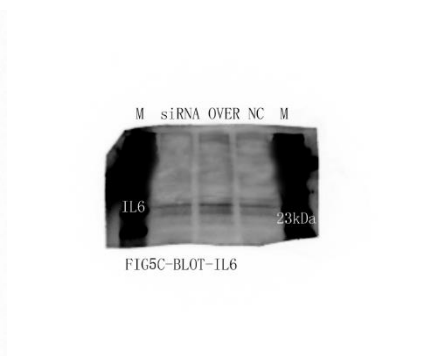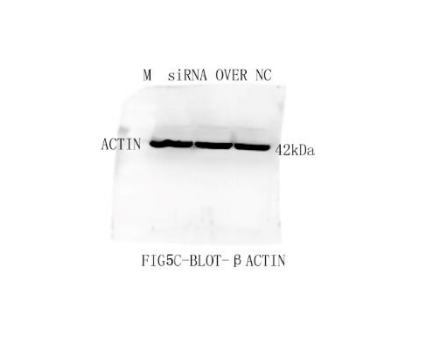

Supplement: Supplemental Material [file KADI_A_2010901_SM9119.zip › supplementary/downloadFromZipFile.pdf]
